# Protective Effects of *Lindera obtusiloba* Leaf Extract on Osteoarthritis in Mouse Primary Chondrocytes and a Medial Meniscus Destabilization Model

**DOI:** 10.3390/ijms26209877

**Published:** 2025-10-10

**Authors:** Kang-Il Oh, Mun Hyoung Bae, Junhwan Jeong, Seokjin Hwang, Jonggyu Park, Hyun-Woo Kwon, Eunkuk Park, Seon-Yong Jeong

**Affiliations:** 1 Department of Medical Genetics, Ajou University School of Medicine, Suwon 16499, Republic of Korea; kyl213@ajou.ac.kr (K.-I.O.); mara24@ajou.ac.kr (M.H.B.); enung7014@ajou.ac.kr (J.J.); tjrwlshh@naver.com (S.H.); 2 BK21 R&E Initiative for Advanced Precision Medicine, Department of Biomedical Sciences, Ajou University Graduate School of Medicine, Suwon 16499, Republic of Korea; 3 Department of Biological Research Laboratory, Jeonbuk Institute for Food-Bioindustry, Jeonju 54810, Republic of Korea; gyu@jif.re.kr (J.P.); hwkwon@jif.re.kr (H.-W.K.)

**Keywords:** osteoarthritis, destabilization of medial meniscus-induced osteoarthritis, *Lindera obtusiloba* leaf, anti-inflammatory effect, herbal medicine

## Abstract

Osteoarthritis (OA) is a degenerative joint disorder characterized by progressive articular cartilage degradation, leading to pain, stiffness, and impaired mobility. This study investigated the anti-osteoarthritic effects of *Lindera obtusiloba* (LO) leaf extract in primary cultured chondrocytes and a mouse model of destabilization of the medial meniscus (DMM)-induced OA. Mouse primary chondrocytes were treated with IL-1β and various concentrations of LO leaf extract (50–150 μg/mL), and analyzed by RT-PCR, Western blotting, and ELISA. For the in vivo experiments, male C57BL/6 mice underwent DMM surgery and were administered LO leaf extract (50–200 mg/kg/day) for eight weeks, followed by micro-CT, histological, and immunohistochemical analyses. LO leaf extract exhibited no cytotoxicity in chondrocytes. In interleukin-1β-induced inflammatory chondrocytes, LO leaf extract significantly suppressed the expression of OA-associated catabolic factors, including cyclooxygenase-2 (Cox-2), matrix metalloproteinases (MMP3 and MMP13), and phosphorylated nuclear factor-kappa B (NF-κB). It also reduced the production of destructive mediators, such as prostaglandin E_2_ (PGE_2_) and collagenase, in a dose-dependent manner. In vivo, LO leaf extract-treated mice demonstrated significant reductions in articular cartilage degradation, subchondral bone sclerosis, and the expression of catabolic and inflammatory mediators. Additionally, LO leaf extract administration significantly decreased systemic pro-inflammatory cytokine levels in DMM-induced mice. Collectively, these findings indicate that LO leaf extract attenuates OA progression by suppressing both local and systemic inflammatory responses, supporting its potential as a natural therapeutic agent for the prevention and treatment of OA.

## 1. Introduction

Osteoarthritis (OA) is a prevalent degenerative joint disorder characterized by pain, stiffness, and structural alterations in the knee joint, affecting tissues, such as the synovium, ligaments, and articular cartilage [1]. Recent global estimates indicate that over 600 million individuals are affected by OA, with the knee being the most commonly involved joint [2]. Factors, such as intense physical activity, injuries, repetitive kneeling, obesity, and genetic predisposition increase mechanical stress on the knee, resulting in articular cartilage deterioration [3].

The knee joint is composed of multiple key components, such as the articular cartilage, menisci, and supporting ligaments [4]. The articular cartilage is a highly specialized connective tissue with a smooth, white, and glossy appearance that serves as a protective layer covering the surfaces of adjacent bones [5]. Composed of approximately 80% water, its extracellular matrix—rich in collagen and proteoglycans—provides structural stability [6]. The meniscus, a crescent-shaped fibrocartilage located between the femur and tibia, plays a crucial role in stabilizing the knee joint, distributing the load, absorbing mechanical stress, and lubricating the joint [7]. Ligaments, consisting of flexible fibrous connective tissue, protect joints from stress-induced damage [8]. These components maintain joint stability and enable the knee to function under significant mechanical load [9].

Repeated or excessive kneeling can induce joint instability, thereby contributing to the development of subchondral bone sclerosis and pathological alterations in periarticular tissues [10,11]. OA is a whole-joint disease that affects all joint tissues, including the synovial membrane and infrapatellar fat pad, which function together as an anatomo-morphological unit and become inflamed and fibrotic during disease progression [12]. The infrapatellar fat pad plays a crucial role in knee biomechanics and contributes significantly to joint pathology [13]. Damaged joint cells, such as chondrocytes, synovial fibroblasts, monocytes, macrophages, and adipocytes from the infrapatellar fat pad, secrete inflammatory cytokines, including interleukin-1β (IL-1β) [14,15,16,17]. IL-1β binds to its receptor (IL-1R) on osteoarthritic chondrocytes, initiating a signaling cascade involving inhibitory kappa B (IκB) kinases [18]. Activated IκB kinase phosphorylates and degrades IκB, enabling nuclear factor-kappa B (NF-κB) to translocate to the nucleus, resulting in increased inflammatory gene expression [19,20,21]. Moreover, external stress–induced IL-1β activates several branches of the mitogen-activated protein kinase (MAPK) cascade, including the ERK, JNK, and p38 pathways, thereby amplifying downstream inflammatory signaling [15]. Increased MAPK activation has been consistently observed in patients with OA, contributing to the upregulation of matrix metalloproteinases (MMPs), enzymes that degrade articular cartilage [22]. In chondrocytes, stimulation by IL-1β and activation of NF-κB signaling promote the production of key catabolic mediators associated with OA, including cyclooxygenase-2 (COX-2), MMP-3, MMP-13, prostaglandin E_2_ (PGE_2_), and several pro-inflammatory cytokines such as interferon-gamma (IFN-γ), IL-1β, IL-6, and TNF-α [23]. The upregulation of these molecules drives cartilage matrix degradation and accelerates the progression of OA [24].

Current OA treatments primarily focus on reducing inflammation, a central feature of OA pathogenesis [25]. Nonsteroidal anti-inflammatory drugs (NSAIDs), including COX inhibitors such as dexamethasone, meloxicam, and aspirin, are widely prescribed to relieve pain and suppress PGE_2_ production by attenuating inflammatory pathways in chondrocytes [26]. However, long-term use of these drugs is limited by adverse effects on the kidneys, heart, gastrointestinal tract, and liver, which substantially increase the overall risks associated with prolonged NSAID therapy [27].

In contrast, medicinal plants have been traditionally used as alternative remedies, offering pain-relieving and anti-inflammatory benefits with generally fewer side effects compared to conventional synthetic agents [28,29]. *Lindera obtusiloba* (LO), belonging to the Lauraceae family, is widely distributed in East Asia, parts of America, and tropical regions. It has traditional uses in herbal medicine, fragrance production, and as a source of essential oils [30]. LO leaf extract exerts beneficial effects both in vitro and in vivo, including ameliorative effects on coagulation and thrombotic activities [31] and protection against oxidative stress-induced liver damage [32]. Additionally, LO leaf extract has demonstrated anti-inflammatory effects in various models, including preadipocytes [33], allergy [34], and dermatitis [35]. Despite its promising effects, the use of LO leaf extract for OA treatment remains unexplored.

In this study, we aimed to understand the protective effects of LO leaf extract on OA by analyzing its effects on catabolic factors and destructive mediators in primary cultured chondrocytes in vitro. Additionally, we assessed the inhibitory effects of LO leaf extract on subchondral sclerosis, histological alterations, and inflammatory cytokine production in a mouse model of destabilization of medial meniscus (DMM)-induced OA.

## 2. Results

### 2.1. LO Leaf Extract Attenuates IL-1β-Induced Inflammatory Responses

We first assessed its cytotoxicity in primary cultured mouse chondrocytes. Cells were treated with LO leaf extract at concentrations of 50, 100, and 150 µg/mL for 48 h, and cell viability was determined using the water-soluble tetrazolium salt assay. The results demonstrated that LO leaf extract did not exert cytotoxic effects at the tested concentrations (Figure S1). Next, we investigated the potential anti-inflammatory effects of LO leaf extract in all three concentrations. Inflammation in primary mouse chondrocytes is primarily triggered by the upregulation of IL-1β, a primary pro-inflammatory cytokine that facilitates the expression of catabolic enzymes involved in cartilage degradation [36].

**Figure 1:**
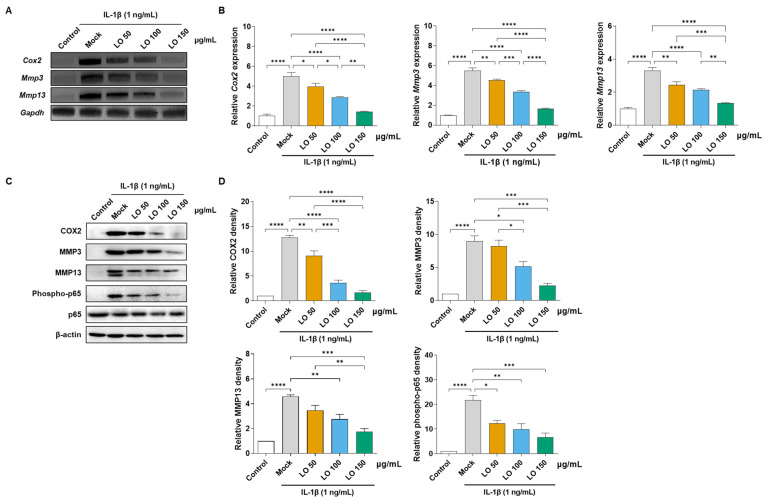
Anti-inflammatory effects of *Lindera obtusiloba* (LO) leaf extract on interleukin-1 beta (IL-1β)-stimulated catabolic responses in mouse primary chondrocytes. Mouse primary chondrocytes were exposed to IL-1β (1 ng/mL) with or without co-treatment with LO leaf extract (50, 100, or 150 μg/mL) for 48 h. (**A**) Transcript levels of cyclooxygenase-2 (*Cox2*), metalloproteinase 3 (*Mmp3*), and *Mmp13* were examined by RT-PCR, and (**B**) further assessed by quantitative real-time PCR. (**C**) Western blot analysis was performed to evaluate COX2 and phosphorylated NF-κB p65 in cell lysates, as well as MMP3 and MMP13 in culture supernatants. β-actin was used as a loading control. (**D**) Densitometric analysis of band intensities for COX2, MMP3, MMP13, and phospho-NF-κB p65. Data are expressed as the mean ± standard deviation of three independent experiments. One-way analysis of variance (ANOVA) was performed, followed by Tukey’s honest significant difference post hoc test (* *p* < 0.05, ** *p* < 0.01, *** *p* < 0.001, **** *p* < 0.0001).

To assess the anti-inflammatory effects of LO leaf extract, chondrocytes were stimulated with IL-1β (1 ng/mL) and co-treated with LO leaf extract at concentrations of 50, 100, and 150 µg/mL for 48 h. The mRNA expression levels of primary catabolic and inflammatory mediators, such as *Cox2*, *Mmp3*, and *Mmp13*, were quantified using RT-PCR and qRT-PCR. IL-1β stimulation significantly increased the mRNA expression of *Cox2*, *Mmp3*, and *Mmp13*, whereas co-treatment with LO leaf extract suppressed mRNA expression in a dose-dependent manner, with significant inhibition observed at higher concentrations (100 and 150 μg/mL) (Figure 1A,B). Western blot analysis confirmed that LO leaf extract (100 and 150 μg/mL) significantly attenuated the protein expression of COX2, MMP3, and MMP13, and phosphorylated NF-κB p65, a crucial mediator of inflammatory signaling (Figure 1C,D). In summary, these results indicate that LO leaf extract mitigates IL-1β-induced inflammatory responses in primary chondrocytes without inducing cytotoxic effects.

### 2.2. LO Leaf Extract Reduces IL-1β-Induced Secretion of PGE_2_ and Collagenase in Primary Chondrocytes

**Figure 2:**
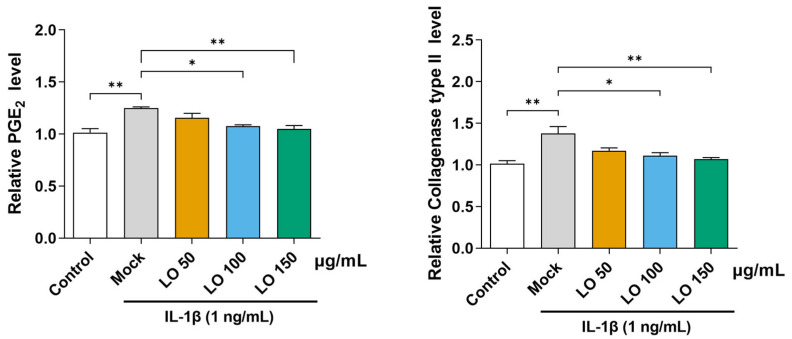
Suppressive effects of *Lindera obtusiloba* (LO) leaf extract on prostaglandin E_2_ (PGE_2_) and collagenase release in IL-1β-stimulated primary chondrocytes. Primary mouse chondrocytes were exposed to IL-1β (1 ng/mL) with or without co-treatment with LO leaf extract (50, 100, or 150 μg/mL) for 48 h. The levels of secreted PGE_2_ and collagenase in the culture supernatants were measured using enzyme-linked immunosorbent assay after concentrating the medium. Data are expressed as the mean ± standard deviation of at least three independent experiments. One-way analysis of variance (ANOVA) was performed, followed by Tukey’s honest significant difference post hoc test (* *p* < 0.05, ** *p* < 0.01).

We assessed the anti-inflammatory effects of LO leaf extract on the secretion of catabolic mediators (PGE_2_ and collagenase) in IL-1β-stimulated mouse primary chondrocytes. Chondrocytes were treated with IL-1β (1 ng/mL) and co-incubated with LO leaf extract at concentrations of 50, 100, and 150 μg/mL for 48 h. After the incubation, the cell culture supernatants were collected and concentrated using Vivaspin^®^ centrifugal concentrators. Subsequently, the levels of PGE_2_ and collagenase were quantified using enzyme-linked immunosorbent assay. IL-1β significantly increased the secretion of both inflammatory mediators PGE_2_ and collagenase. Treatment with LO leaf extract markedly reduced IL-1β-induced PGE_2_ and collagenase secretion, with significant inhibition observed at higher concentrations (100 and 150 μg/mL) (Figure 2).

### 2.3. LO Leaf Extract Reduces Subchondral Sclerosis in a DMM-Induced OA Mouse Model

**Figure 3:**
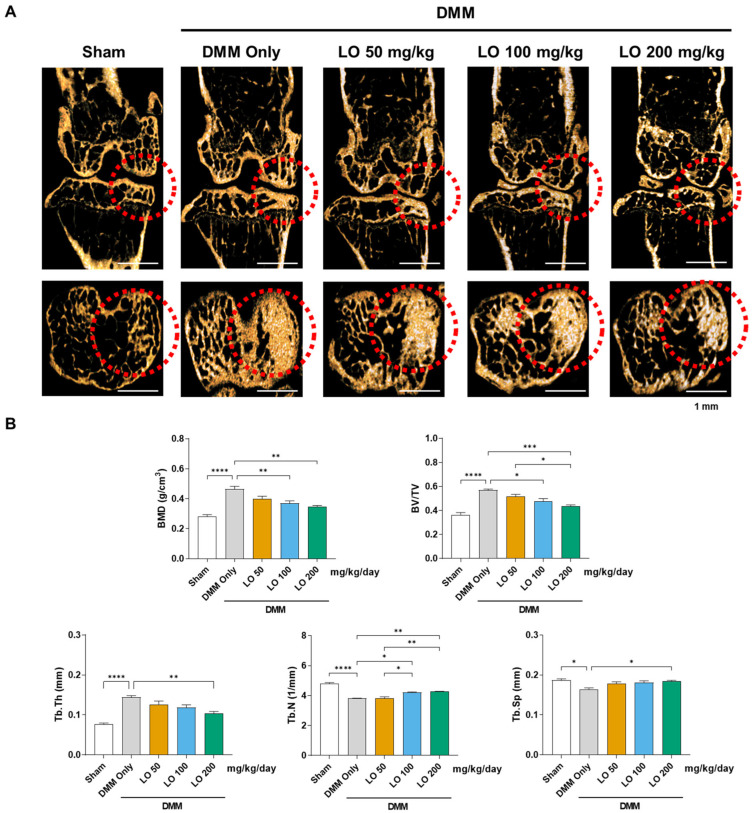
Effects of *Lindera obtusiloba* (LO) leaf extract on subchondral bone remodeling in a destabilization of the medial meniscus (DMM)-induced mouse model of osteoarthritis (OA). OA was induced in mice by DMM surgery, followed by oral administration of LO leaf extract at the indicated doses (50, 100, and 200 mg/kg/day) for eight weeks. (**A**) Representative micro-computed tomography images of the left knee joints showing subchondral bone structure. Scale bar: 1 mm. (**B**) Quantitative analysis of bone mineral density (BMD), bone volume fraction (BV/TV), trabecular thickness (Tb.Th), trabecular number (Tb.N), trabecular separation (Tb.Sp) in the subchondral region of interest. Data are expressed as mean ± standard deviation. One-way analysis of variance (ANOVA) was performed, followed by Tukey’s honest significant difference post hoc test (* *p* < 0.05, ** *p* < 0.01, *** *p* < 0.001, **** *p* < 0.0001).

To assess the in vivo protective effects of LO leaf extract against OA, a DMM mouse model was used. Male C57BL/6 mice underwent DMM surgery and were subsequently administered LO leaf extract-supplemented diets at doses of 50, 100, or 200 mg/kg/day for eight weeks. At the end of the treatment period, the operated left knee joints were harvested and subjected to micro-computed tomography (micro-CT) analysis. DMM surgery resulted in increased bone mineral density (BMD) and bone volume fraction (BV/TV) in the subchondral bone, accompanied by greater trabecular thickness (Tb.Th) and reduced trabecular number (Tb.N) and trabecular separation (Tb.Sp), collectively indicating subchondral sclerosis. Notably, dietary administration of LO leaf extract (100 and 200 μg/mL) significantly attenuated these DMM-induced bone structural alterations in the subchondral region, demonstrating that LO leaf extract effectively mitigates subchondral bone sclerosis associated with OA progression (Figure 3A,B).

### 2.4. Histological Assessment of Cartilage Protection by LO Leaf Extract in a DMM-Induced OA Mouse Model

**Figure 4:**
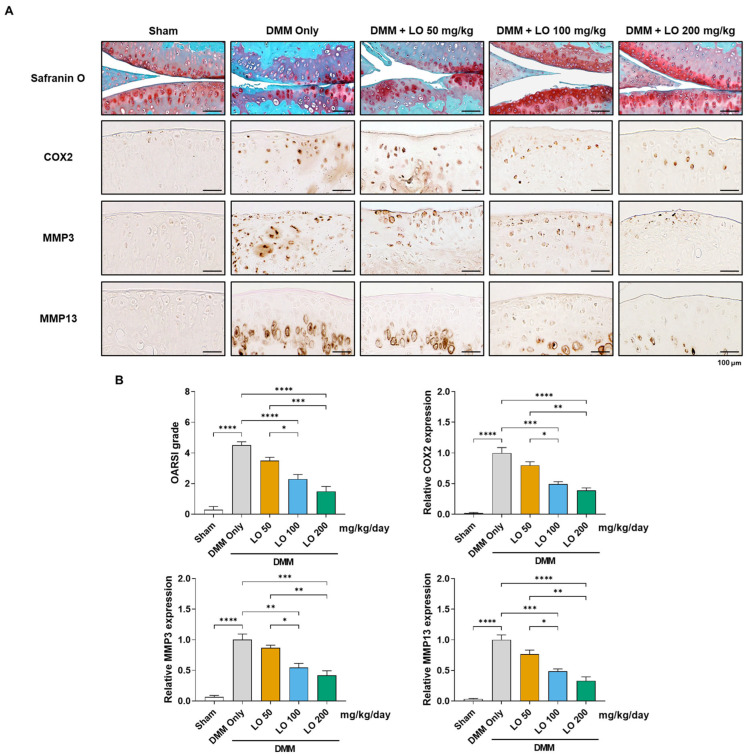
Protective effects of *Lindera obtusiloba* (LO) leaf extract on articular cartilage degradation and catabolic factor expression in mice with destabilization of the medial meniscus (DMM)-induced osteoarthritis (OA). (**A**) Histological evaluation of mouse knee joints subjected to DMM surgery and orally administered LO leaf extract (50, 100, or 200 mg/kg/day) for eight weeks, including Safranin O staining and immunohistochemistry for COX2, MMP3, and MMP13 in the articular cartilage. Scale bar: 100 µm. (**B**) Quantitative assessment of cartilage degeneration using the Osteoarthritis Research Society International (OARSI) scoring system and semiquantitative assessment of catabolic marker expression levels. Data are presented as mean ± standard deviation. One-way analysis of variance (ANOVA) was performed, followed by Tukey’s honest significant difference post hoc test (* *p* < 0.05, ** *p* < 0.01, *** *p* < 0.001, **** *p* < 0.0001).

To confirm the chondroprotective effects of LO leaf extract on cartilage destruction, histological analysis was conducted in the DMM-induced OA mouse model. DMM-operated mice exhibited significant cartilage degradation in the operated knee joint, characterized by a significant reduction in Safranin O staining and increased expression of catabolic markers compared with those in the sham-operated group. Immunohistochemical analysis revealed significant upregulation of COX2, MMP3, and MMP13 in the articular cartilage of the DMM group. In contrast, LO leaf extract-treated mice exhibited a dose-dependent reduction in cartilage destruction and suppressed expression of these catabolic enzymes (Figure 4A), with significant inhibition observed at higher concentrations (100 and 200 μg/mL) (Figure 4B). Cartilage structural integrity was further assessed using the Osteoarthritis Research Society International (OARSI) scoring system, which revealed a significant reduction in DMM-induced cartilage erosion and vertical clefts extending to the calcified cartilage zone in the LO leaf extract-treated groups (100 and 200 μg/mL) (Figure 4B). These results indicate that LO leaf extract reduces cartilage degeneration in OA by attenuating inflammatory and catabolic processes in the joint microenvironment.

### 2.5. Modulatory Effect of LO Leaf Extract on Systemic Inflammatory Cytokines in a DMM-Induced OA Mouse Model

**Figure 5:**
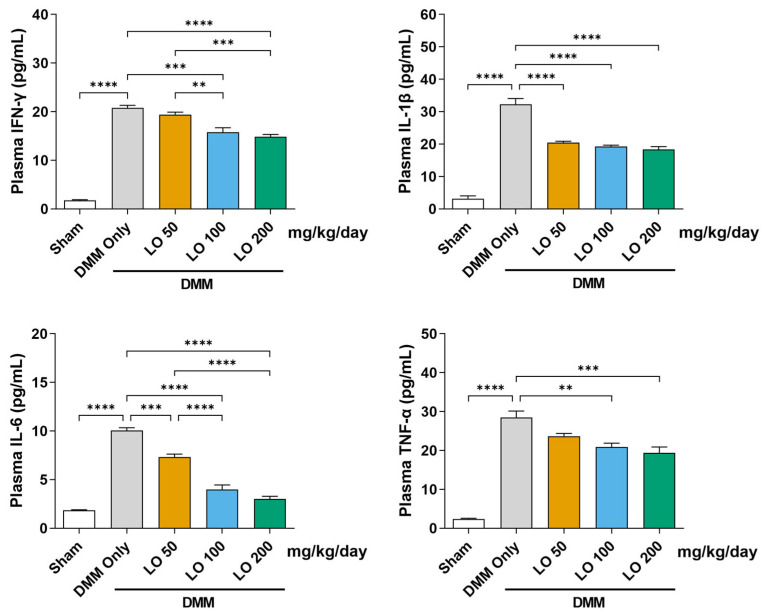
Effects of *Lindera obtusiloba* (LO) leaf extract administration on systemic inflammatory cytokine levels in a mouse with destabilization of the medial meniscus (DMM)-induced osteoarthritis (OA). Plasma concentrations of inflammatory mediators, including interferon-γ (INF-γ), IL-1β, IL-6, and TNF-α were quantified in DMM-induced OA mice orally administered LO leaf extract at various doses (50, 100, and 200 mg/kg/day) for eight weeks. Cytokine levels were measured using enzyme-linked immunosorbent assay. Data are presented as mean ± standard deviation. One-way analysis of variance (ANOVA) was performed, followed by Tukey’s honest significant difference post hoc test (** *p* < 0.01, *** *p* < 0.001, **** *p* < 0.0001).

Systemic anti-inflammatory effects of LO leaf extract were evaluated by measuring plasma cytokine levels in mice with DMM-induced OA. Compared with the sham group, DMM-operated mice (DMM only) exhibited significantly elevated levels of IFN-γ, IL-1β, IL-6, and TNF-α. Oral administration of LO leaf extract at 100 and 200 mg/kg/day for eight weeks significantly suppressed these pro-inflammatory cytokines in the plasma (Figure 5). These results indicate that LO leaf extract mitigates OA-associated systemic inflammation by downregulating the circulating levels of pro-inflammatory cytokines, potentially contributing to the overall amelioration of OA pathology.

## 3. Discussion

OA is a chronic, progressive joint disease characterized by structural and functional deterioration of the synovial joints, involving pathological alterations in articular cartilage, subchondral bone, synovial membrane, and periarticular tissues that contribute to joint dysfunction, chronic pain, and disability [37,38]. As the most common form of arthritis worldwide, OA imposes a substantial public health and socioeconomic burden, particularly among aging populations in which its prevalence is steadily increasing [39]. Importantly, OA is now recognized as a whole-joint disease rather than a cartilage-limited disorder, with pathological changes extending across multiple joint tissues [38]. Persistent low-grade inflammation is now recognized as a primary contributor to disease progression and pain sensitization in OA, driven by disrupted tissue homeostasis, increased activity of cartilage-degrading enzymes, oxidative stress, and sustained production of pro-inflammatory mediators [36,40]. Current treatments, such as NSAIDs and corticosteroids, offer symptomatic relief but fail to prevent disease progression—often causing serious side effects, including gastrointestinal, renal, and cardiovascular complications [41,42]. Despite advances in understanding the pathogenesis of OA, no effective disease-modifying OA drugs are currently available, highlighting a critical unmet medical need [41]. These limitations highlight the urgent need for safer, disease-modifying therapies, with plant-derived natural products emerging as promising alternatives because of their multi-targeted actions, low toxicity, and ability to modulate both local joint pathology and systemic inflammation [43]. Therefore, this study assessed the protective effects of LO leaf extract on OA progression. While our IL-1β-stimulated model at 1 ng/mL provided valuable insights into the inflammatory responses of chondrocytes, we acknowledge that alternative inflammation models—such as TNF-α stimulation or macrophage-conditioned medium—may offer a more physiologically relevant representation of the complex inflammatory microenvironment in OA [44,45]. This limitation will be addressed in future studies by employing these models to further validate the protective effects of LO leaf extract.

The present study demonstrates that LO leaf extract exerts potent anti-inflammatory activity in primary mouse chondrocytes by attenuating IL-1β-induced activation of major inflammatory signaling factors. In the context of OA pathogenesis, IL-1β acts as a central mediator that activates the NF-κB and MAPK pathways, thereby upregulating catabolic factors—including COX-2, MMP-3, and MMP-13—that contribute to cartilage matrix degradation [18,36]. Consistent with previous findings that botanical compounds can inhibit IL-1β-induced inflammatory cascades and preserve cartilage integrity [43,46], LO leaf extract significantly downregulated the expression of COX2, MMP3, and MMP13 at both the mRNA and protein levels. Additionally, it significantly reduced the secretion of downstream catabolic mediators (PGE_2_ and collagenase) in IL-1β-stimulated chondrocytes. These mediators contribute to cartilage matrix degradation and joint inflammation, making their suppression crucial for OA treatment [47]. Our results are consistent with previous reports demonstrating that phytochemicals with anti-inflammatory properties inhibit prostaglandin synthesis by downregulating COX-2 activity, thereby counteracting the progression of OA [48,49].

DMM-induced OA mice typically develop subchondral bone sclerosis with OA progression caused by abnormal mechanical stress and osteoblast hyperactivity [50,51]. In our in vivo experiment, micro-CT analysis revealed that LO leaf extract treatment significantly prevented the DMM-induced increase in subchondral BMD, suggesting a protective effect against aberrant subchondral bone remodeling during OA progression. This observation consistent with previous reports demonstrating that attenuation of subchondral bone alterations can slow OA progression and improve overall joint integrity [52,53]. Additionally, histological assessment confirmed the cartilage-protective effects of the LO leaf extract. Mice treated with LO leaf extract exhibited significantly lower OARSI scores and reduced expression of catabolic enzymes, including MMP-3, MMP-13, and COX-2, in the articular cartilage. As MMP-13 is significantly involved in type II collagen degradation and MMP-3 facilitates the activation of other matrix-degrading enzymes [54,55], their downregulation indicates that LO leaf extract preserves the cartilage structure by disrupting the catabolic cascade. Plant-derived compounds with anti-inflammatory and antioxidant effects can effectively reduce cartilage degradation in surgically induced OA models [56,57].

Notably, OA is increasingly recognized as a systemic inflammatory disease, with increased circulating cytokines, such as IL-1β, IL-6, TNF-α, and IFN-γ, contributing to local joint pathology, pain sensitization, and systemic inflammation [16,58,59]. Our results demonstrate that LO leaf extract significantly reduced serum pro-inflammatory cytokine levels, indicating potential immunomodulatory effects on immune and inflammatory responses. This systemic anti-inflammatory action supports previous studies that natural products can restore immune homeostasis and suppress chronic inflammation at both local and systemic levels [60,61,62].

Quercetin was identified as a primary bioactive ingredient of the LO leaf extract; however, the present study was designed to evaluate the therapeutic potential of the total extract rather than the isolated compound. This approach reflects the understanding that herbal extracts typically contain multiple phytochemicals that may act synergistically to produce enhanced therapeutic effects. While quercetin is a major flavonoid with well-documented anti-inflammatory and chondroprotective properties, other constituents are also likely to contribute to the observed protective effects [63]. Therefore, we focused on assessing the biological activity of the total extract in this study. Nonetheless, we acknowledge that a more detailed characterization of additional constituents is required. Future studies will investigate the specific contributions of quercetin and other bioactive compounds to the overall activity of the LO leaf extract, providing deeper insight into potential synergistic or additive mechanisms. In addition, we plan to perform comprehensive mass spectrometry analyses to identify and characterize the complete phytochemical profile of the LO leaf extract, which will yield valuable insights into possible synergistic interactions among its various bioactive constituents.

In summary, these results indicate that LO leaf extract reduces OA progression by attenuating local joint inflammation, structural damage, and systemic inflammatory responses, supporting its potential as a safe and effective natural therapeutic agent for OA management. This study provides evidence that LO leaf extract exerts protective effects against OA progression by modulating primary molecular and structural alterations during articular cartilage degradation. Using chondrocyte cultures and DMM-induced mice, the LO leaf extract demonstrated the ability to suppress inflammatory signaling and reduced joint tissue damage. Collectively, our findings indicate that LO leaf extract may serve as a complementary therapeutic agent for the management of OA, potentially enhancing the efficacy of existing clinical treatments, as reported for other plant-derived compounds that act synergistically with conventional anti-inflammatory therapies [64].

## 4. Materials and Methods

### 4.1. Extraction and Identification of Quercitrin from LO Leaf Extract

Dried LO leaves, grown in Korea, were purchased from the Cheongmyeung Herb (Chungju, Chungcheongbuk-do, Republic of Korea; http://www.good1075.com). For extraction, dried LO leaves (100 g) were added to 30% ethanol (2000 mL, 20 times the weight of leaves) and extracted at 70 °C for 8 h. The resulting solution was filtered through an 11 nm membrane and the filtrate was concentrated to a final Brix value of 20–25 using rotary evaporation at 50 °C. The concentrated extract (28 g) was subsequently freeze-dried. For cell-based experiments, the freeze-dried LO leaf extract was dissolved in distilled water to prepare stock solutions, which were then diluted to the desired concentrations in culture medium immediately before use. Quercitrin was identified using gradient elution with a solvent system of 0.1% trifluoroacetic acid in water and acetonitrile at 5, 20, 25, and 40%, and two steps of 5% acetonitrile. High-performance liquid chromatography was performed using an Agilent system (Agilent Technologies, Santa Clara, CA, USA) equipped with a Hypersil GOLD™ column (Thermo Fisher Scientific, Waltham, MA, USA). The chromatographic conditions included a column temperature of 25 °C, detection at 280 nm, and flow rate of 0.6 mL/min. The presence of quercitrin, a primary bioactive marker of LO, was confirmed by its retention time and UV absorbance profile (Figure S2A,B).

### 4.2. Primary Culture of Chondrocytes from Mouse Knee Cartilage

Primary chondrocytes were harvested from the knee articular cartilage of 5-day-old Institute of Cancer Research (ICR) mice (DBL Co., Ltd., Chungbuk, Republic of Korea). Cartilage tissues were dissected and digested in Dulbecco’s modified Eagle’s medium (DMEM; Invitrogen, Carlsbad, CA, USA) containing 1% collagenase type II (Sigma-Aldrich, St. Louis, MO, USA) and 0.5% trypsin-EDTA for 2 h at 37 °C, following a previously described protocol [65]. After discarding the initial digestion supernatant, the remaining cartilage fragments were subjected to a second digestion with 1% collagenase type II in DMEM for an additional 2 h at 37 °C.

The resulting cell suspension was neutralized in DMEM supplemented with 10% fetal bovine serum and 1% antibiotic–antimycotic solution (Invitrogen). The mixture was then filtered through a 40 μm Falcon^®^ cell strainer (Corning, NY, USA) and centrifuged at 1200 rpm for 2 min to harvest chondrocytes. To establish an inflammatory model, the isolated chondrocytes were stimulated with IL-1β (GenScript, Piscataway, NJ, USA) for 48 h.

### 4.3. Water-Soluble Tetrazolium Salt Assay

Primary mouse chondrocytes were plated in 96-well culture plates at a density of 1 × 10^5^ cells per well and maintained for 72 h at 37 °C in a humidified incubator containing 5% CO_2_. Following incubation, the cells were exposed to LO leaf extract at concentrations of 50, 100, and 150 µg/mL for 48 h. Cell viability was determined using a D-Plus™ CCK assay kit (Donginbiotech, Seoul, Republic of Korea) in accordance with the manufacturer’s instructions. Absorbance values were subsequently recorded at 450 nm using a microplate reader (Bio-Rad, Hercules, CA, USA).

### 4.4. Quantitative Real-Time Polymerase Chain Reaction Analysis

Primary mouse chondrocytes were seeded in 35 mm culture dishes at a density of 3 × 10^5^ cells and maintained at 37 °C in a humidified incubator containing 5% CO_2_ for 72 h. Cells were then stimulated with IL-1β (1 ng/mL) alone or in combination with LO leaf extract (50, 100, or 150 µg/mL) for 48 h. Total RNA was extracted using TRIzol reagent (Invitrogen, Carlsbad, CA, USA) according to the manufacturer’s protocol. RNA concentration and purity were determined by measuring the absorbance ratio at 260/280 nm. Complementary DNA (cDNA) was synthesized from 1 µg of total RNA using the RevertAid™ H Minus First Strand cDNA Synthesis Kit (Fermentas, Hanover, NH, USA).

For gene expression analysis, reverse transcription PCR was carried out using HiPi Plus 5X PCR MasterMix (ELPIS Biotech, Daejeon, Republic of Korea), and quantitative real-time PCR (qRT-PCR) was performed using the SYBR Green I qPCR kit (TaKaRa, Shiga, Japan) on a CFX Connect Real-Time PCR Detection System (Bio-Rad, Hercules, CA, USA). The primer sequences used for qRT-PCR are listed in Table 1.

The qRT-PCR cycling conditions consisted of an initial denaturation at 95 °C for 5 min, followed by 44 cycles of denaturation at 95 °C for 5 s, annealing at 60 °C for 20 s, and extension at 72 °C for 30 s. A melt curve was generated from 72 °C to 95 °C, with 1 °C increments at 5 s intervals. Relative gene expression levels were normalized to *Gapdh* and quantified using the 2^−ΔΔCt^ method.

### 4.5. Western Blot Analysis

Cells were lysed in radioimmunoprecipitation assay (RIPA) buffer (BIOSESANG, Seongnam, Gyeonggi-do, Republic of Korea) supplemented with phenylmethylsulfonyl fluoride (Abcam, Cambridge, UK) and a protease inhibitor cocktail (Sigma-Aldrich). Protein concentrations were quantified using the Lowry assay. The cell lysates were mixed with 5× sodium dodecyl sulfate (SDS) loading buffer (BIOSESANG), boiled at 100 °C for 5 min. Equal amounts of protein (20 μg per lane) were separated by 12% SDS-polyacrylamide gel electrophoresis and transferred onto polyvinylidene fluoride (PVDF) membranes. The membranes were blocked with 5% bovine serum albumin (Bovogen, Melbourne, Australia) for 1 h at room temperature with gentle agitation. Subsequently, the membranes were incubated overnight at 4 °C with the following primary antibodies, diluted according to the manufacturers’ recommendations: anti-COX2 (1:1000; sc-1745; Santa Cruz Biotechnology, Dallas, TX, USA), anti-MMP3 (1:1000; ab52915; Abcam), anti-MMP13 (1:1000; ab51072; Abcam), anti-NF-κB p65 (1:1000; #8242; Cell Signaling Technology, Danvers, MA, USA), phospho-NF-κB p65 (1:1000; Ser536; ab86299; Abcam), and anti-β-actin (1:1000; sc-47778; Santa Cruz Biotechnology). Band intensities were quantified using ImageJ software (Version 1.53g; National Institutes of Health, Bethesda, MD, USA).

### 4.6. PGE_2_ and Collagenase Quantification

The concentrations of PGE_2_ and collagenase released into the culture supernatants were quantified using a Prostaglandin E2 Parameter Assay Kit (KGE004B; R&D Systems, Minneapolis, MN, USA) and EnzChek™ Gelatinase/Collagenase Assay Kit (E12055; Invitrogen), respectively. Prior to measurement, the culture supernatants were concentrated using a Vivaspin^®^ 2 Centrifugal Concentrator (Sartorius, Göttingen, Germany).

### 4.7. DMM Mouse Model

All animal experiments were conducted in accordance with the guidelines of the Institutional Animal Care and Use Committee (IACUC) of Ajou University (Approval No. 2020-0036) and adhered to the institutional ethical standards. Male C57BL/6 mice (9 weeks old) were obtained from DBL Co., Ltd. (Eumseong, Republic of Korea) and housed under controlled environmental conditions (23 ± 2 °C, 55 ± 5% humidity, and 12 h light/dark cycle) with free access to standard chow and water. OA was induced in the left knee joint using the DMM surgical model performed under anesthesia with tiletamine/zolazepam (Zoletil; Virbac Laboratories, Carros, France). Following surgery, the animals received daily oral administration of LO leaf extract at doses of 50, 100, or 200 mg/kg for 8 weeks via medicated feed. Each experimental group consisted of six mice (*n* = 6), and the sample size was determined based on earlier reports employing the DMM model [50,51]. At the end of the experimental period, the mice were euthanized, and blood samples were collected for plasma isolation and stored at –80 °C. The operated knee joints were fixed in 4% paraformaldehyde (BIOSESANG) at 4 °C for subsequent histological and biochemical analyses.

### 4.8. Micro-CT Analysis

BMD of the operated knee joints was analyzed using high-resolution micro-CT (SkyScan 1272; Bruker, Billerica, MA, USA). Scans were acquired in a spiral mode under the following parameters: 60 kV X-ray voltage, 400 μA current, 360° rotation, and image acquisition with a charge-coupled device camera (1280 × 1280 resolution) at a 400 ms exposure time. Regions of interest were defined within the meniscal destabilization-affected zone of the articular cartilage. The data were reconstructed and analyzed using CTVox software version 3.2 (Bruker).

### 4.9. Histology (Safranin O Staining and Immunohistochemistry)

After fixation, the knee joints were decalcified in 0.5 M EDTA (pH 8.0) for 2 weeks, embedded in paraffin, and sectioned at 6 μm thickness using a rotary microtome (Leica, Wetzlar, Germany). For immunohistochemical analysis, tissue sections were deparaffinized in xylene (3 times for 5 min each) and rehydrated through a graded ethanol series (99.9%, 95%, and 70%) to distilled water. Endogenous peroxidase activity was quenched with 3% H_2_O_2_ for 10 min followed by phosphate-buffered saline (PBS) washes (3 times for 5 min each). Antigen retrieval was performed using Tris-EDTA buffer (pH 9.0) at 97 °C for 10 min. Sections were then blocked with 1% BSA in PBS and washed with PBS (3 times for 5 min each). Primary antibodies against COX2, MMP3, and MMP13 (diluted 1:100) were applied and incubated overnight at 4 °C. After washing with PBS containing 0.1% Tween-20 (PBS-T) (3 times for 5 min each), sections were incubated with secondary antibody for 1 h at room temperature. Following additional PBS-T wases (3 times, 5 min each), immunoreactivity was visualized with 3,3′-diaminobenzidine (DAB) for 1 min, washed with PBS (3 times for 5 min each), and counterstained with Mayer’s hematoxylin for 1 min. The stained slides were visualized and digitized using the Axio Scan.Z1 slide scanner (Carl Zeiss, Oberkochen, Germany). Cartilage destruction was graded using the OARSI scoring system [66].

### 4.10. Cytokine Analysis

Plasma levels of pro-inflammatory cytokines (IFN-γ, IL-1β, IL-6, and TNF-α) were determined using a multiplex bead-based immunoassay (Mouse Cytokine/Chemokine Magnetic Bead Panel; MCYTOMAG-70K, Millipore, Billerica, MA, USA). The assays were carried out according to the manufacturer’s instructions, and data were acquired using the MAGPIX^®^ multiplexing system (Luminex Corporation, Austin, TX, USA).

### 4.11. Statistical Analysis

Quantitative data are shown as means ± standard deviation (SD) from at least three independent experiments. Comparisons between two groups were performed using an unpaired Student’s *t*-test, while differences among multiple groups were assessed using one-way analysis of variance (ANOVA) followed by Tukey’s post hoc test. The normality of data distribution was evaluated using the Shapiro–Wilk test, and variance homogeneity was verified with Levene’s test, confirming that all datasets met the assumptions required for parametric analyses. Statistical analyses were performed using GraphPad Prism software (version 9.2.0; GraphPad Software, San Diego, CA, USA) and SPSS software (version 11.0; SPSS Inc., Chicago, IL, USA). A probability value (*p*-value) < 0.05 was considered statistically significant. No pre-established inclusion or exclusion criteria were applied. All animals (*n* = 6 per group) completed the study and were included in all analyses without exclusions. All histological evaluations and quantitative analyses were conducted by assessors blinded to the group assignments.

## Figures and Tables

**Table 1:** Primer sequences used for quantitative real-time PCR.

Gene	Forward Primer (5′ → 3′)	Reverse Primer (5′ → 3′)
*Gapdh*	TCA CTG CCA CCC AGA C	TGT AGG CCA TGA GGT CCA C
*Cox2*	GGT CTG GTG CCT GGT CTG ATG AT	GTC CTT TCA AGG AGA ATG GTG C
*Mmp3*	CTG TGT GTG GTT GTG TGC TCA TCC TAC	GGC AAA TCC GGT GTA TAA TTC ACA ATC
*Mmp13*	TGA TGG ACC TTC TGG TCT TCT GGC	CAT CCA CAT GGT TGG GAA GTT CTG

## Data Availability

The original contributions presented in this study are included in the article/Supplementary Materials. Further inquiries can be directed to the corresponding authors.

## Supplementary Materials

The following supporting information can be downloaded at https://www.mdpi.com/article/10.3390/ijms26209877/s1.

## Abbreviations

The following abbreviations are used in this manuscript:

BMD	bone mineral density
COX2	cyclooxygenase II
DMEM	Dulbecco’s modified Eagle’s medium
DMM	destabilization of medial meniscus
EDTA	ethylenediaminetetraacetic acid
IκB	inhibitory kappa B
IL	interleukin
IFN-γ	interferon gamma
LO	*Lindera obtusiloba*
MAPK	mitogen-activated protein kinase
micro-CT	micro-computed tomography
MMPs	metalloproteinases
NF-κB	nuclear factor-kappa B
NSAIDs	non-steroidal anti-inflammatory drugs
OA	osteoarthritis
OARSI	Osteoarthritis Research Society International
PGE_2_	prostaglandin E2
TNF-α	tumor necrosis factor-α
