# Supplementary material for: Protective Effects of Lindera obtusiloba Leaf Extract on Osteoarthritis in Mouse Primary Chondrocytes and a Medial Meniscus Destabilization Model
Source: Int J Mol Sci. 2025 Oct 10;26(20):9877. doi: 10.3390/ijms26209877 (PMC12564646; doi:10.3390/ijms26209877)

Supplementary Data

Protective effects of *Lindera obtusiloba* leaf extract on osteoarthritis in mouse primary chondrocytes and a medial meniscus destabilization model

Kang-Il Oh [1,2,3,†], Mun Hyoung Bae [1,2,†], Junhwan Jeong [1,2,3], Seokjin Hwang [1,2], Jonggyu Park [4], Hyun-Woo Kwon [4], Eunkuk Park [1,4,*], Seon-Yong Jeong [1,2,3,*]

1 Department of Medical Genetics, Ajou University School of Medicine, Suwon 16499, Republic of Korea; kyl213@ajou.ac.kr (K.-I.O.); mara24@ajou.ac.kr (M.H.B.); enung7014@ajou.ac.kr (J.J.); tjrwlshh@naver.com (S.H.)
2 Department of Biomedical Sciences, Ajou University Graduate School, Suwon 16499, Republic of Korea
3 BK21 R&E Initiative for Advanced Precision Medicine, Department of Biomedical Sciences, Ajou University Graduate School, Suwon 16499, Republic of Korea
4 Department of Biological Research Laboratory, Jeonbuk Institute for Food-Bioindustry, Jeonju 54810, Republic of Korea; gyu@jif.re.kr (J.P.); hwkwon@jif.re.kr (H.-W.K.)
* Correspondence: ekpark@jif.re.kr (E.P.); jeongsy@ajou.ac.kr (S.-Y.J.)
† These authors have contributed equally.

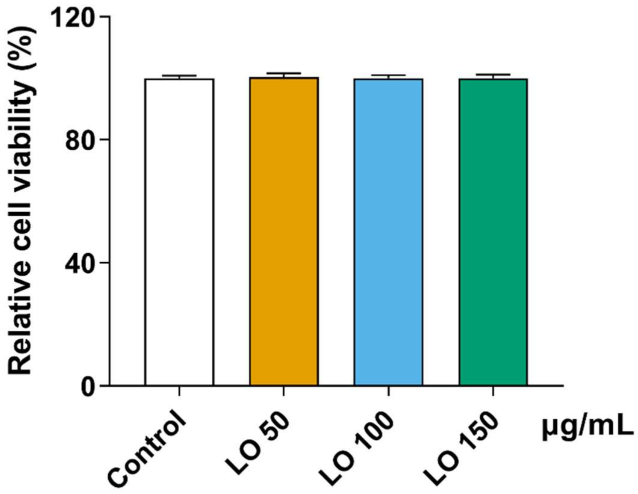

Figure S1. Effects of *Lindera obtusiloba* (LO) leaf extract on the cytotoxicity of mouse primary chondrocytes. Mouse primary cultured chondrocytes were incubated with three different concentrations of LO leaf extract (50, 100, and 150 μg/mL) for 48 h. A water-soluble tetrazolium salt assay was performed to assess cytotoxicity in mouse primary chondrocytes.

A

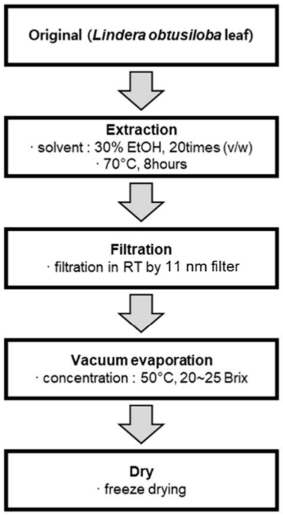

Conditions	Contents
Column	Thermo Scientific Hypersil GOLD™ (4.6 x 250 mm, 5 μm)
Column temperature	25°C
Mobile phase A	Water with 0.1% Trifluoroacetic acid
Mobile phase B	Acetonitrile
Mobile phase gradient	
Injection volume	10 μL
Detection wavelength	280 nm
Flow rate	0.6 mL/min

Mobile phase gradient:

Time (min)	Solvent A (%)	Solvent B (%)
0	95	5
5	80	20
10	75	25
20	60	40
10	95	5
20	95	5

B

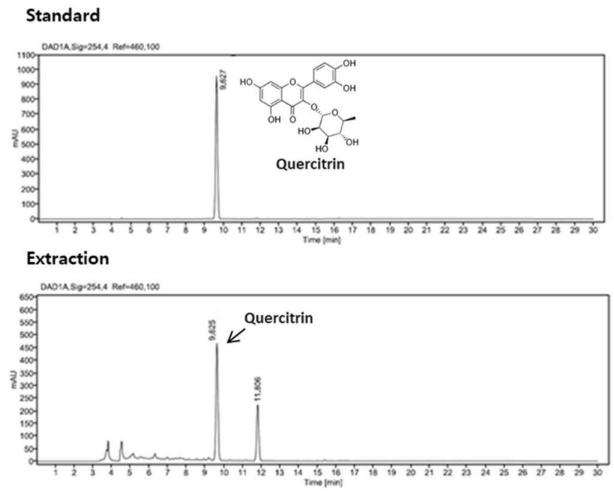

Figure S2. Isolation and identification of quercitrin from *Lindera obtusiloba* (LO) leaf extract. **(A)** Schematic representation of the extraction process and high-performance liquid chromatography (HPLC) conditions used for the 30% ethanol extract of LO leaves. **(B)** Detection and confirmation of quercitrin in the LO extract using HPLC analysis.

Raw Western Blotting Data

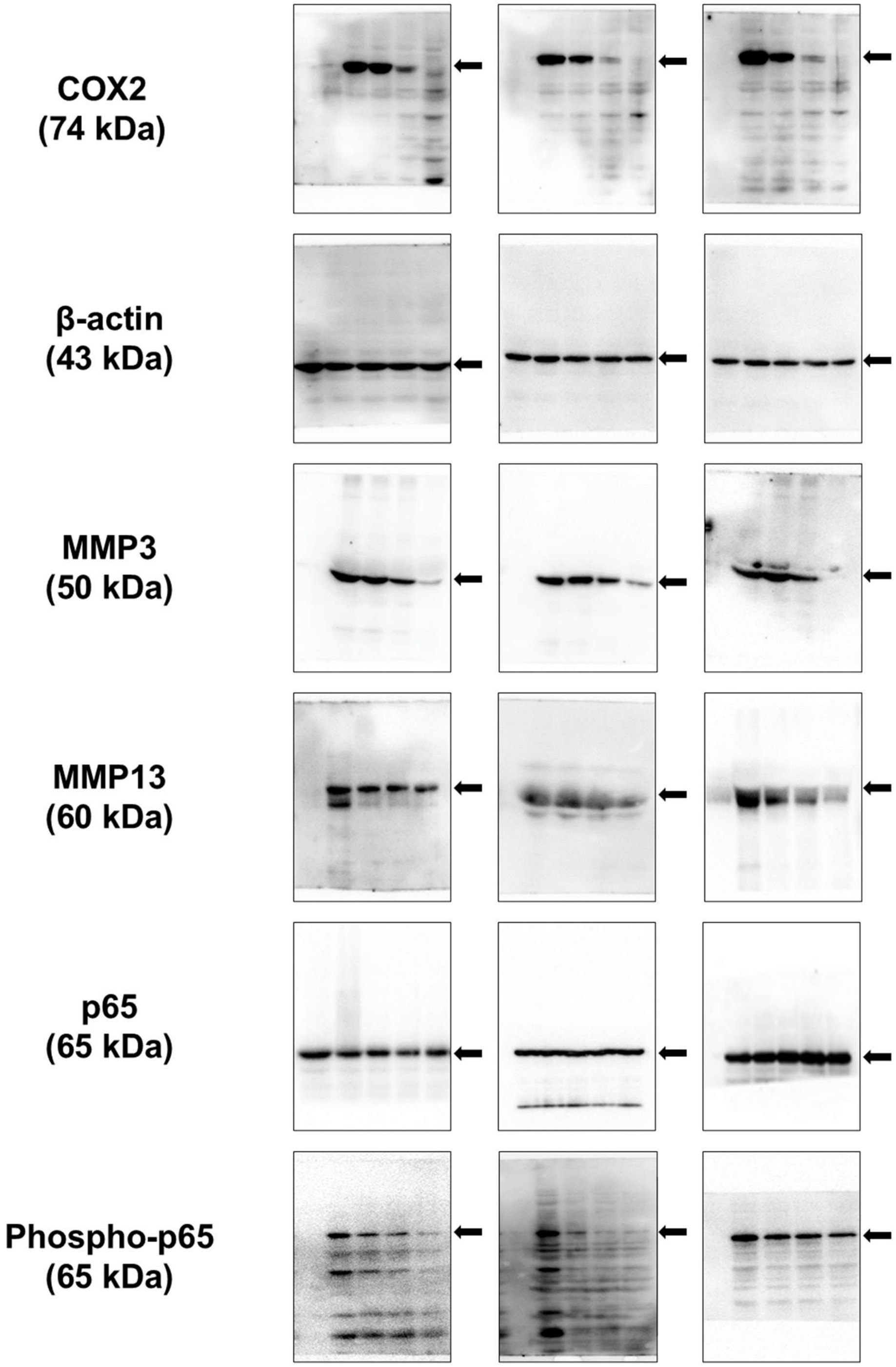

Supplement: Supplementary file 1 [file ijms-26-09877-s001.zip › ijms-3893175-supplementary.pdf]
